# Differential effects of Losartan and Atorvastatin in partial and full thickness burn wounds

**DOI:** 10.1371/journal.pone.0179350

**Published:** 2017-06-14

**Authors:** Johanneke J. Akershoek, Katrien M. Brouwer, Marcel Vlig, Bouke K. H. L. Boekema, Rob H. J. Beelen, Esther Middelkoop, Magda M. W. Ulrich

**Affiliations:** 1 Department of Plastic, Reconstructive and Hand Surgery, Research Institute MOVE, VU University Medical Center, Amsterdam, The Netherlands; 2 Association of Dutch Burn Centres, Beverwijk, The Netherlands; 3 Department of Molecular Cell Biology and Immunology, VU University Medical Center, Amsterdam, The Netherlands; National Centre for Scientific Research-Demokritos, GREECE

## Abstract

Healing of burn wounds is often associated with scar formation due to excessive inflammation and delayed wound closure. To date, no effective treatment is available to prevent the fibrotic process. The Renin Angiotensin System (RAS) was shown to be involved in fibrosis in various organs. Statins (e.g. Atorvastatin), Angiotensin receptor antagonists (e.g. Losartan) and the combination of these drugs are able to reduce the local RAS activation, and reduced fibrosis in other organs. We investigated whether inhibition of the RAS could improve healing of burn wounds by treatment with Atorvastatin, Losartan or the combination of both drugs. Therefore, full and partial thickness burn wounds were inflicted on both flanks of Yorkshire pigs. Oral administration of Atorvastatin, Losartan or the combination was started at post-burn day 1 and continued for 28 days. Full thickness wounds were excised and transplanted with an autologous meshed split-thickness skin graft at post-burn day 14. Partial thickness wounds received conservative treatment. Atorvastatin treatment resulted in enhanced graft take and wound closure of the full thickness wounds, faster resolution of neutrophils compared to all treatments and reduced alpha-smooth muscle actin positive cells compared to control treatment. Treatment with Losartan and to a lesser extent the combination therapy resulted in diminished graft take, increased wound contraction and poorer scar outcome. In contrast, Losartan treatment in partial thickness wounds decreased the alpha-smooth muscle actin^+^ fibroblasts and contraction. In conclusion, we showed differential effects of Losartan and Atorvastatin in full and partial thickness wounds. The extensive graft loss seen in Losartan treated wounds is most likely responsible for the poor clinical outcome of these full thickness burn wounds. Therefore, Losartan treatment should not be started before transplantation in order to prevent graft loss. Atorvastatin seems to accelerate the healing process in full thickness wounds possibly by dampening the pro-inflammatory response.

## Introduction

Wound healing is a complex and tightly regulated process. The underlying mechanisms leading to full regeneration of the skin or fibrosis (scar formation) are still not clear. Deep partial and full thickness burn wounds often result in hypertrophic scars as a result of a derailment of the healing process. Scars, especially located at joints, are a burden for patients due to functional limitations in joint mobility, and they often need multiple reconstructive surgeries [1].

New therapeutic application areas for existing drugs are an alternative and fast way to develop new treatment strategies for different types of disorders including fibrosis. Angiotensin receptor antagonists and statins are drugs that have been on the market for many decades and were shown to be successful in reducing fibrosis in different organs [2–5]. The original therapeutic goal of angiotensin receptor antagonists is treatment of hypertension by inhibition of the systemic renin angiotensin system (RAS). Statins have been developed to lower the cholesterol level by inhibition of the enzyme 3-hydroxy-3-methylglutaryl coenzyme A reductase, an enzyme involved in cholesterol synthesis. Both drug classes have similar pleiotropic effects; they were shown to have anti-fibrotic and anti-inflammatory effects for fibrotic pathologies in heart [4], kidney [3], liver [5], lung and skin [2].

Burn wounds distinguish themselves from other wounds by an extensive and prolonged pro-inflammatory state, with increased levels of neutrophils, components of the complement system and C reactive protein [6] leading to massive fibrosis. Therefore these drugs might be effective in burn wound healing as well.

**Fig 1:**
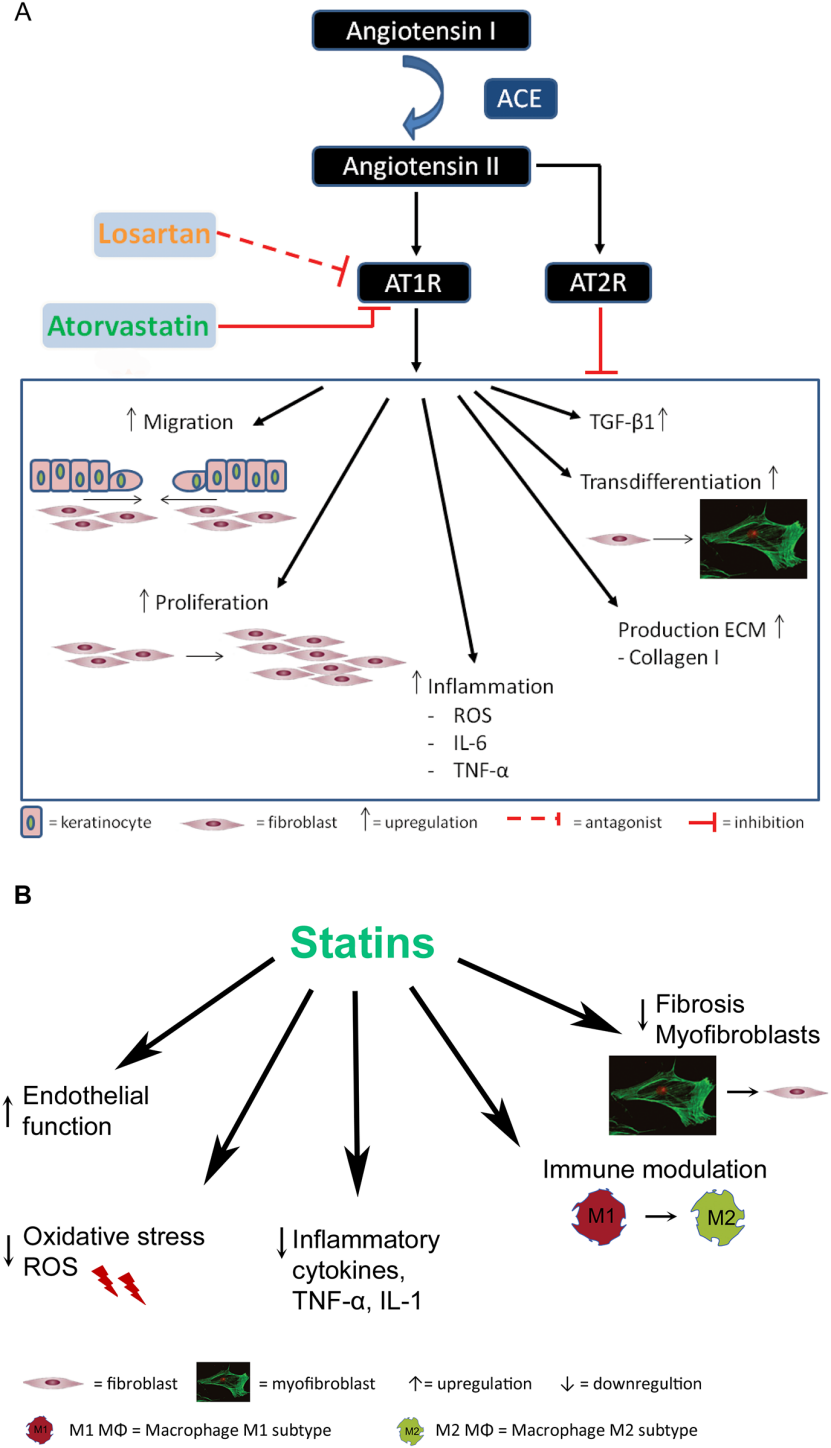
Possible pleiotropic effect of RAS inhibition and statins in wound healing. **Fig 1A. Role of RAS in fibrosis**. AngII is a biologically active component of the RAS which is able to induce several pro-fibrotic actions upon activation of AT1. AngII has been shown to enhance keratinocyte and fibroblast migration and fibroblast proliferation. Furthermore, downstream signaling of the AT1 leads to increased production of TGF-β1 and extracellular matrix (ECM) components and seems to play a role in transdifferentiation of fibroblasts into myofibroblasts. In addition, AT1 activation induces inflammation by upregulation of several inflammatory cytokines, chemokines and growth factors [48–50]. **Fig 1B. Pleitropic effects of statins**. Besides their role in reducing cholesterol levels Statins have been shown to exert several pleiotropic effects. The different actions of statins are mediated by different routes in which the Peroxisome proliferator-activated receptors and the Rho/ROCK pathway play an important role. Statins were shown to reduce the AngII receptor AT1 (fig 1A), modulate the immune reaction and reduce the inflammation. In addition they reduce oxidative stress and improve endothelial cell function [18, 21, 22, 51]. ACE, Angiotensin Converting Enzyme; AngII, Angiotensin II; AT1, Angiotensin 1 receptor; AT2, Angiotensin 2 receptor; IL-6, Interleukin 6; ROS, Reactive Oxygen Species; TGF-β1, Transforming Growth Factor-1; TNF-α, Tumor Necrosis Factor-alpha.

The anti-fibrotic and/or anti-inflammatory effects of angiotensin receptor antagonists are believed to be mainly due to their effect on the tissue renin angiotensin system (tRAS). This local tRAS is present in nearly all organs and tissues of the body including heart, lungs, kidneys, liver and has also been described in the skin [7]. The local functions of tRAS in different tissues have been described by Paul et al. [8]. Overall it plays a role in tissue homeostasis, e.g. cell growth, proliferation and metabolism. (t)RAS consists of several components including the biologically active ligand Angiotensin II (AngII). This ligand can bind to angiotensin receptors 1 (AT1) and/or 2 (AT2) and induce downstream effects (Fig 1). tRAS becomes activated upon tissue damage which starts during the inflammatory and granulation phases of wound healing [9]. Upregulation of both Angiotensin receptor subtypes has been demonstrated after injury of blood vessels, brain, heart, nerves [10] and skin [10, 11]. Chronic tissue injury and the presence of reactive oxygen species (ROS) are thought to extend tRAS activation resulting in the onset of fibrosis [9]. Fibrosis is characterized by excessive synthesis and diminished degradation of extracellular matrix (ECM) proteins such as collagen.

tRAS is thought to regulate fibrosis by the downstream effects of AT1 activation by AngII. Activated AT1 induces transforming growth factor beta-1 (TGF-β1) production, collagen synthesis, cell proliferation (e.g. fibroblasts), migration and inflammation (e.g. upregulation of IL-6) [10]; processes known to be involved in the development of fibrosis. TGF-β1 plays a crucial role in the fibrotic pathologies of all tissue types. Increased TGF-β1 levels contribute to fibrosis by inducing myofibroblast transition. Myofibroblasts are characterized by excessive production of ECM components and the alpha-smooth muscle actin (αSMA) containing stress fiber formation which contributes to contraction of the tissue [10].

The other AngII receptor, AT2, is described as an anti-fibrotic receptor through counteracting the activities of AT1 [10, 12, 13]. However, the precise mechanisms behind the functions and counteracting actions of the AT2 are still unclear. Specific inhibition of the AT1 receptor by using AT1 antagonists such as Losartan not only could reduce the fibrotic response due to AT1 inhibition but could also enhance the anti-fibrotic (and regenerative) functions of the AT2.

In addition to AT1 antagonists, similar anti-fibrotic actions have been described for statins. Statins are mainly known for potent inhibition of the mevalonate pathway, reducing cholesterol levels. However, they also have other pleiotropic effects e.g. immunomodulatory effects and interaction with RAS. Statins were shown to reduce AT1 expression and signaling [14, 15], to inhibit several AngII-dependent intracellular signaling pathways [16], and to diminish RAS-induced reactive oxygen species (ROS) and inflammation [14, 15, 17]. In addition to the interactions with the RAS, statins were shown to exert effects on other pathways and processes (Fig 1B) [18]. Statins modulate the inflammatory response by reducing the recruitment of inflammatory cells and expression of inflammatory mediators such as C—reactive protein, TNFα and interleukin-6. [19–21]. It was shown that Atorvastatin induces the anti-inflammatory M2 macrophage differentiation [22]. The different actions of statins are mediated by different routes in which the Peroxisome proliferator-activated receptors and the Rho/ROCK pathway play an important role [18].

Both drug types have been shown to exert anti-fibrotic and anti-inflammatory effects when given separately. The combination of an AT1 antagonist and a statin, in different rat models, has been shown to have synergistic effects in heart [4, 23–25], kidney and skeletal muscle fibrosis [26]. These studies on combination therapy reported that the enhanced anti-fibrotic effects were mediated by reducing inflammation by lowering C-reactive protein levels, reducing numbers of ED1-positive (pro-inflammatory) macrophages and by attenuating fibrosis through the reduction of AngII-positive cells and TGF-β1 levels. The anti-fibrotic or anti-inflammatory actions of these drugs have not yet been studied in wound healing and/or scar formation in burn wounds. Therefore, the aim of our study was to investigate whether administration of the AT1 antagonist Losartan or the statin Atorvastatin could improve the quality of healing of burn wounds in a porcine model. We chose to evaluate the effects of approved drugs on the healing of burn wounds, because this would allow faster implementation in the clinic when proven beneficial.

A combination therapy of Losartan and Atorvastatin was applied to study the proposed synergistic effects in these wounds.

## Materials and methods

### Animal experiments

**Fig 2:**
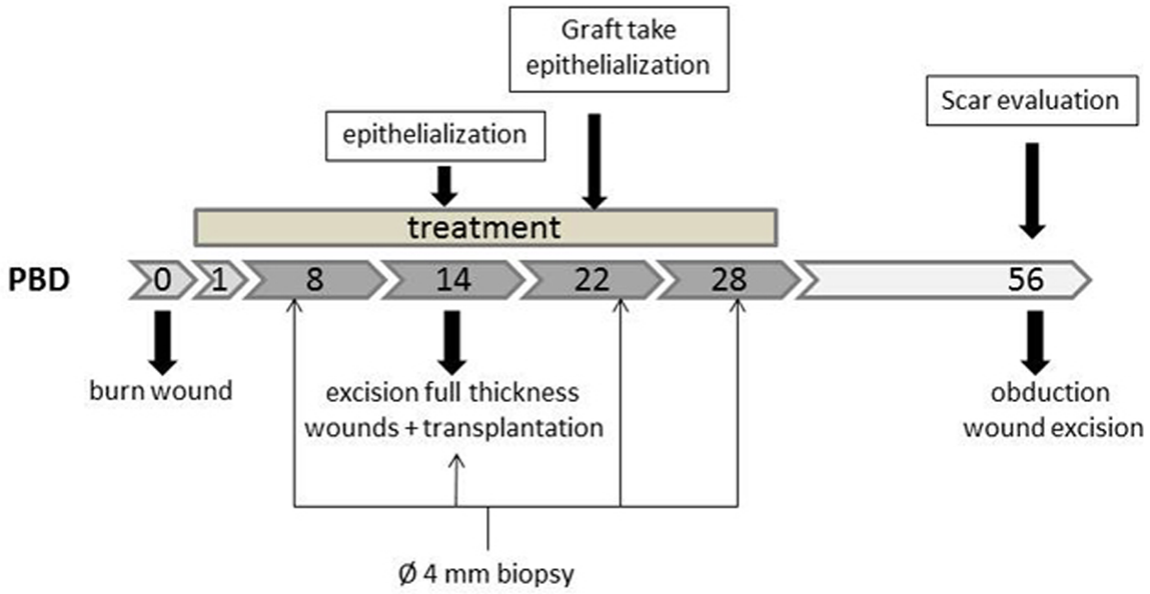
Overview experimental timeline. Treatment with Atorvastatin, Losartan or combination therapy started at PBD 1 and was continued until PBD 28. Full thickness wounds were excised and transplanted with an autologous split-thickness skin graft (STSG) at PBD 14. Skin biopsies were taken at several time points during the experiment, indicated in the scheme above.

The institutional Animal Experiments Committee of the VU University Medical Center Amsterdam, the Netherlands approved the experimental protocols according to governmental and international guidelines on animal experimentation. This experiment was conducted with four female Yorkshire pigs (SPF Pig breeder, van Beek BV, Lelystad, The Netherlands). An adaptation period of two weeks was applied before the start of the experiment. The pigs were individually housed, fed twice a day and had access to water *ad libitum*. At the start of the experiment the body weight of the pigs was on average 37 kg and this reached an average of 61 kg at PBD 56, the end of the experiment. An overview of the experimental schedule is presented in Fig 2.

### Surgical procedure

Sedation of the animals was induced approximately twenty minutes before the start of each procedure by intramuscular (i.m.) injection of a combination of Ketamine 10 mg/kg, Dormicum (Midazolam) 0.5 mg/kg and Atropine 0.5–1.0 mg. Full anesthesia was induced before the infliction of the wounds and surgery by administration of Etomidate lipidor on effect via intravenous (i.v.) injection (sedation) and Dormicum (Midazolam) 15 mg, as analgesia Fentanyl 200 μg was administered and as muscle relaxant Pavulon (Pancuronium bromide) 6 mg (i.v.). The anesthesia during the procedures was maintained by i.v. injection of Dormicum 0.5 mg/kg/h, Fentanyl 6.5 μg/kg/h and Pavulon 0.27 mg/kg/h. Animals under full anesthesia received artificial respiration with 45–50% O_2_ and 1.5–2.5% Isoflurane. Vital functions such as CO_2_ expiration concentration, blood gas values, heart rate and temperature were monitored during surgical procedures. During biopsy and dressing change procedures the animals were anesthetized via inhalation of 35% O_2_, 65% NO_2_ and 1.5–2.5% Isoflurane. Pre-operative pain relief was provided by i.m. administration of analgesic Novum 20 (Meloxicam, 0.04 mg/kg). For analgesia, Buprenorphine 0.3 mg was administered i.m. directly after the surgical procedures. Post-operative analgesia was administered via an adhesive transdermal patch (Transtec 35^®^), containing 20 mg Buprenorphine, on the abdomen of the animal. This patch was replaced after 3 days and analgesia was continued for 14 days.

A modified procedure of the porcine excisional wound model described by our group [27] was used. In brief, a grid of 8 x 8 cm squares was tattooed on both flanks of the pigs at the day of infliction of the burn wounds. The purpose of this grid was to correct for the growth of the animal in the calculation of contraction. Three full thickness and three partial thickness contact burn wounds of 4 x 4 cm squares were inflicted on each flank of the pig. Burn wounds were induced by placing a brass stamp (160 g) of 170°C for 10 seconds (partial thickness) or 20 seconds (full thickness) on the skin without applying extra pressure. Wounds were covered with adhesive Allevyn (gentle border 10 x 10 cm, Smith&Nephew, Hull, UK) to protect against mechanical trauma and contamination. Sterile gauzes (45 x 70 cm) were used to cover the Allevyn bandages. These were fixed with adhesive bandage Curafix (Lohmann & Rauscher GmbH & Co., Neuwied, Germany) and covered with an elastic stocking Tubigrip (Klinion, Mediq Medeco, Oud-Beijerland, The Netherlands).

At day 14 after infliction of the burn wounds, full thickness wounds were excised with a scalpel until the subcutaneous fat layer (± 2.7 mm deep), followed by transplantation of meshed (expanded to a 1:3 ratio) autologous split-thickness skin graft (STSG). Excision of the wounds and transplantation of the STSG was performed at PBD 14 to correlate with the average timing of escharotomy and transplantation used in the clinical setting in Europe [28]. Autologous STSGs (0.3 mm thickness) were obtained from the dorsum of the pig using an electrical dermatome (Humeca, Enschede, The Netherlands). The harvested skin for the STSG was meshed (Humeca) at a ratio of 1:3 and kept moisturized in sterile phosphate buffered saline (PBS)-soaked gauzes until use. STSGs were transplanted on the excised full thickness burn wounds and fixed with staples (3M, Delft, The Netherlands). Wounds were dressed as described above.

### Treatments

The animals received the following treatments: A) no treatment (standard control treatment), B) Atorvastatin, C) Losartan or D) combination therapy of Atorvastatin and Losartan. Six full thickness wounds and six partial thickness wound were evaluated per treatment. The treatment was started one day after infliction of the burn wound to mimic the clinical routine as much as possible, since patients are usually not immediately admitted to the hospital or burn center after the burn incident. Following clinical human prescription; Atorvastatin was administered in two subsequent dosages; 20 mg day was administered for a period of 14 days, after which the Atorvastatin dose was increased to 40 mg/day for an additional 14 days. Losartan (0.8 mg/kg starting body weight per day) was administered for 28 days. The same dosages of Atorvastatin and Losartan were used for the combination therapy. The drugs were orally administered by mixing pulverized tablets with a small portion of pre-wetted food in a bowl.

### Macroscopic wound evaluation and dressing changes

Digital pictures of the wounds were taken during dressing change at PBD 0, 8, 14, and 22, and just before euthanasia at PBD 56. Macroscopic evaluation of the wounds from the digital pictures was performed by six independent trained observers. Pictures of the wounds were randomized and scored blinded. Wounds were evaluated for graft take and wound closure (re-epithelialization) at day 22 for full thickness wounds (Table 1). At the end of the experiment (PBD 56), each wound was assessed on site for level of scarring compared to normal skin and given an overall observer score for scar quality by two independent trained observers (Table 1). Parameters used in the macroscopic evaluation were adjusted from the observer part of the Patient and Observer Scar Assessment Scale (POSAS), which is a validated method for scar evaluation in patients [29, 30]. This overall observer score (scar quality) was based on scored parameters such as contraction (distortion), scar color (redness), scar thickness. Normal, uninjured skin is scored 10 and the poorest outcome imaginable receives the grade 1. To measure contraction the wound edges and tattoo grid were traced onto a transparent sheet at days 0 and 56. Contraction of the wound was determined by planimetry using the image analysis program NIS Elements (version 3.1, Nikon, Amsterdam, The Netherlands). Contraction of the wound was corrected for the growth of the animal using the tattooed grid as follows: ratio wound area to the area of the tattooed grid area. The ratio wound area/ tattoo grid area is expressed as a percentage. Finally, the ratio at time point 0 is compared with time point 56. The contraction of the wound is calculated by the following formula:

$$wound\;contraction\;\left(\%\right)=\left(1-\frac{\left(ratio\frac{wound\;area\;t_{56}}{tattoo\;grid\;area\;t_{56}}\right)}{\left(ratio\;\frac{wound\;area\;t_{0}}{tattoo\;grid\;area\;t_{0}}\right)}\right)x\;100\%$$

*T*_*56*_ = *day 56*

*t*_*0*_ = *day 0 (no contraction)*

**Table 1:** Parameters macroscopical wound evaluation.

Parameter	Score	Description	PBD
Graft take	Percentage (%) vital graft of total graft area	i.e. viability of the graft (pink color) and adherence to the wound bed (%)	22
Epithelialization	Percentage (%) of total wound area covered by epithelium	Re-epithelialization of the wound area	22,28
Observer score (scar quality)	Scale 1 to 10	compared to unaffected skin (1 = unaffected skin, 10 = poorest outcome imaginable)	56

Punch biopsies (Ø 4 mm) were taken on PBD 8, 14, 22and28 from each wound in a systematic order. From full thickness wounds samples were taken from excised tissue on PBD 14. At the end of the experiment (PBD 56), the animals were euthanized with 30 mL pentobarbital sodium i.v., and subsequently a large biopsy across the center of the wound was taken. Wound biopsies were fixed in Kryofix (48% ethanol, 7% polyethylene glycol (PEG)-300) and stored at 4°C for at least 2 days.

### Microscopic wound evaluation

Fixed tissue biopsies were dehydrated, embedded in paraffin and cut in sections of five μm thickness. For (immuno-)histochemical stainings, sections were deparaffinized and rehydrated. Sections were stained with hematoxylin and eosin (H&E) for histological analysis.

Scoring for presence of an epidermis in the partial thickness wounds was performed manually using a scoring system. The H&E stained slides were blinded and scored by 2 experienced researchers. The scoring was as follows: no epidermis: 0, partial epidermis: 1, complete epidermis: 2.

Presence of myofibroblasts in wound tissue sections was assessed at PBD 56, by αSMA staining using an antibody against αSMA (Clone 1A4, dilution 1:500; Dako, Glostrup, Denmark). Neutrophils were stained at PBD 8, 14, 22 and 28, for full thickness wounds and at PBD 8, 14 and 22 for partial thickness wounds, using an antibody directed against Myeloperoxidase (MPO; Clone A0398, dilution 1:1200; Dako, Glostrup, Denmark).

BrightVision Poly-HRP anti-mouse or anti-rabbit (Immunologic, Duiven, The Netherlands) were used as secondary antibodies for the αSMA and MPO staining respectively. The substrate 3,3'-Diaminobenzidine (DAB, Immunologic) was used to visualize the staining. Tissue sections were counterstained with hematoxylin (Mayers; Dako). Tissue biopsies stained for αSMA expression were digitally scanned with the Aperio Scanscope AT2 (Leica Microsystems B.V, Eindhoven, The Netherlands). Digital images for tissue sections stained for MPO expression were taken with a DXM1200F camera mounted at an Axioskop 40 bright field microscope (Zeiss, Oberkochen, Germany). The αSMA and MPO expression, indicated by the DAB signal, were analyzed by selection of the DAB signal in the tissue using the threshold function in NIS Elements.

The αSMA expression by myofibroblasts was analyzed in tissue sections by using a region of interest (ROI) covering the area between the epidermis and the subcutaneous adipose tissue. First, a threshold was set for the total DAB signal inside the ROI, which was analyzed. Second, to extract the αSMA-expressing blood vessels a threshold was set for the blood vessels inside the ROI based on high intensity and area size of the DAB signal. Blood vessels that express αSMA frequently have a dense and intense DAB signal and often have a larger area size than myofibroblasts. Expression of αSMA by myofibroblasts was calculated by extraction of the measured αSMA-positive blood vessel area from the total αSMA-positive ROI area and expressed as a fraction of the ROI area.

For the analysis of MPO-positive neutrophils in the wound area, the DAB signal was extracted from the hematoxylin signal in the images by using the ImageJ (version 1.49p) plugin “color deconvolution” designed by G. Landini. This plugin color deconvolution is based on the method described by Ruifrok et al. [31]. We used this method in order to threshold only the MPO-positive neutrophils without interference of the hematoxylin signal using NIS Elements. The DAB signal was measured as an area (μm^2^) and calculated as a fraction of the total tissue area (%).

### Statistics

All statistical analyses were performed with SPSS (version 24.0 MS Windows, SPSS Inc, Chicago, IL, USA). Statistically significant differences between treatment groups were determined using a two-tailed Mann-Whitney U test. Statistically significant differences between different time points were determined using Wilcoxon signed rank test. Results are presented in a dot plot, where each dot represents a wound, and the line represents the median. A *p*-value of < 0.05 was considered statistically significant.

## Results

### Acute inflammation

**Fig 3:**
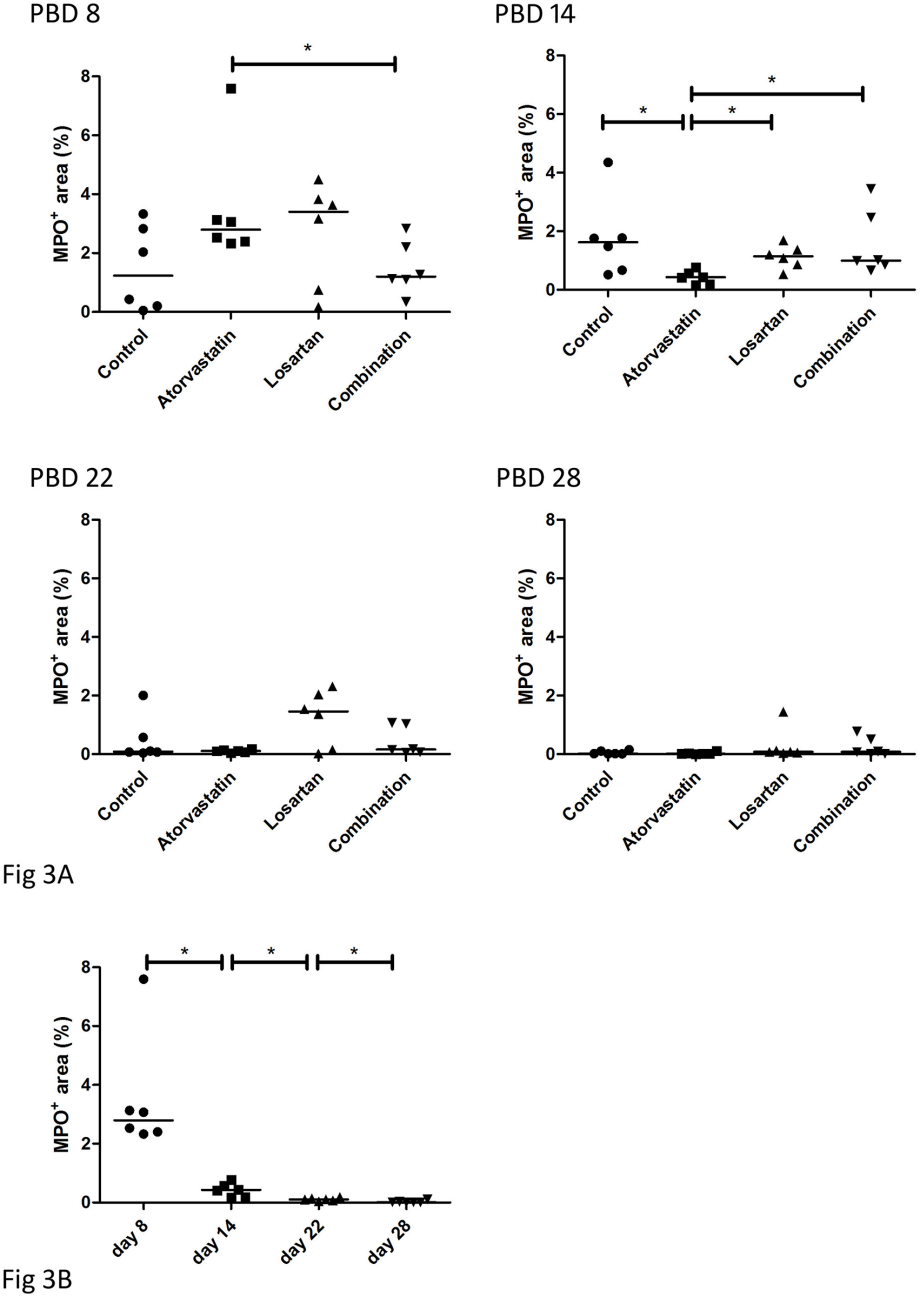
Presence of neutrophils in full thickness wounds. Neutrophils in the wound areas were detected by immunohistochemical staining of paraffin sections using an antibody against myeloperoxidase (MPO), which is expressed mainly by neutrophils. A) MPO positive areas at different time points per treatment. To show the differences between the treatment groups we plotted the same results per time point in B).

**Fig 4:**
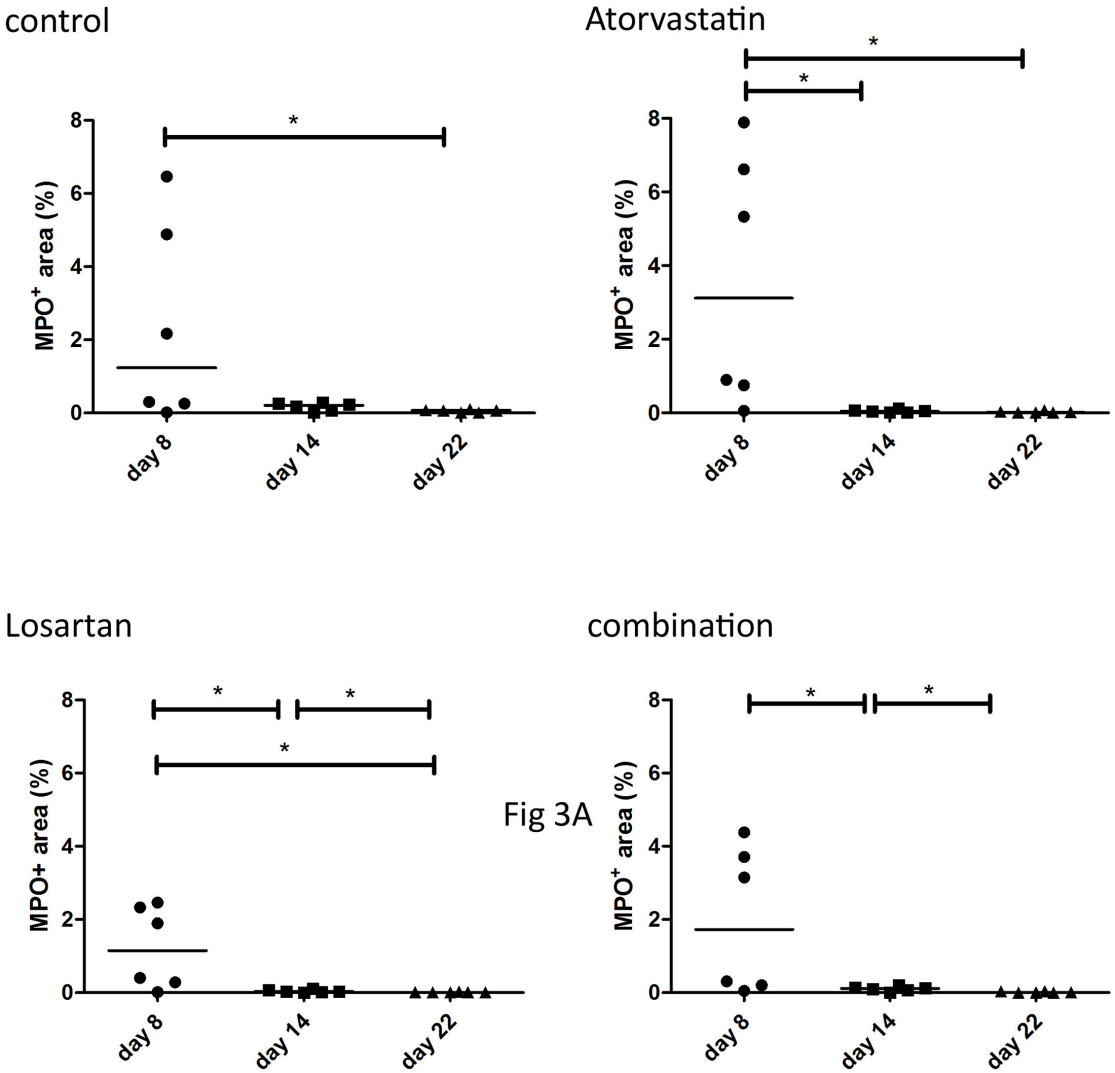
Presence of neutrophils in partial thickness wounds. As in Fig 3A) MPO positive areas at different time points per treatment.

Burn wounds are characterized by a prolonged acute inflammatory response. In this study we analyzed the presence of neutrophils as a marker for the acute inflammation phase. Tissue sections from PBD 8, 14 and 22 and for full thickness wounds also at PBD 28 (Figs 3 and 4) were stained for MPO expression to assess the presence of activated neutrophils. Early during wound healing (PBD 8), the wounds of the animals treated with Atorvastatin or Losartan showed a slightly increased MPO-positive area in the full thickness wounds. However, in the Atorvastatin treated wounds a statistically significant decrease in MPO-positive area was measured during the consecutive days of analysis indicating a faster resolution of the pro-inflammatory phase (Fig 3A) in these full thickness wounds. Moreover, at PBD 14, the day of excision of the wound and subsequent transplantation with the STSG, the Atorvastatin treated wounds showed a significantly reduced MPO expression in comparison to all other groups (Fig 3B).

**Fig 5:**
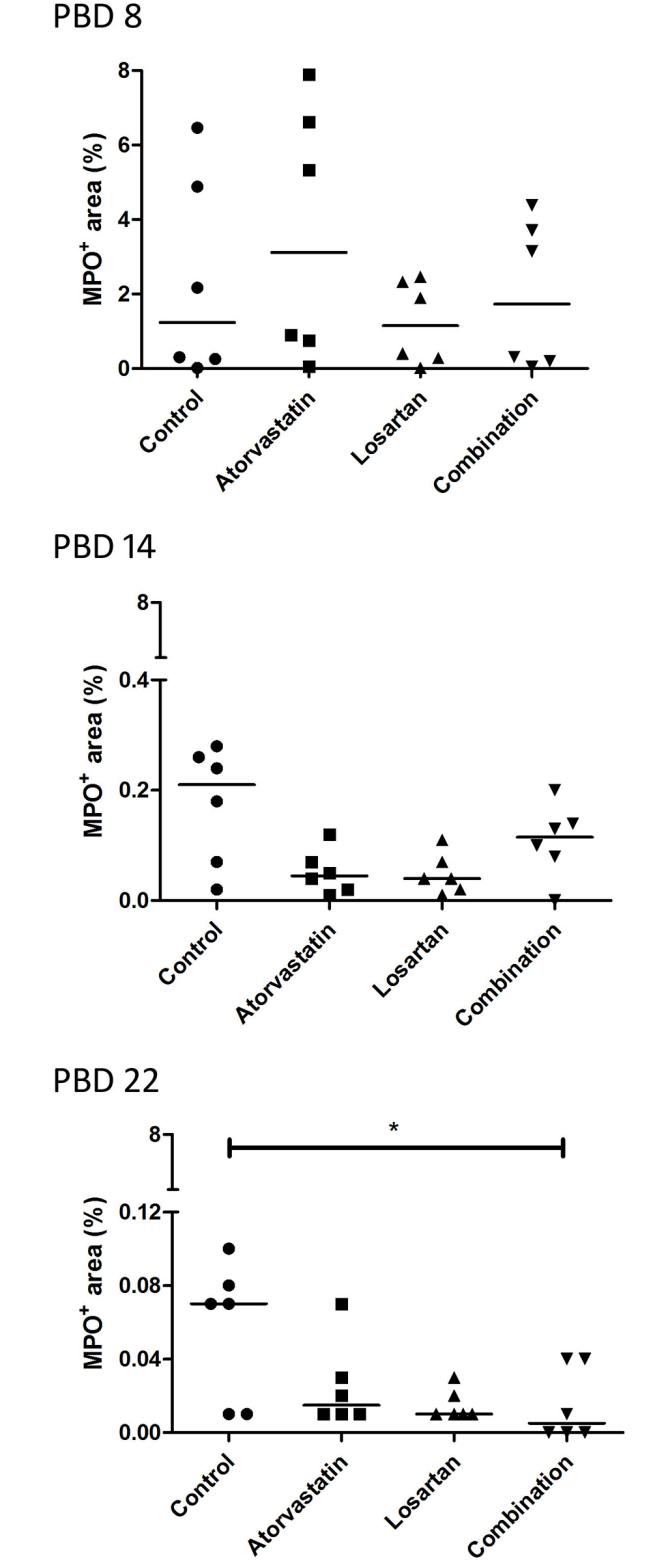
Presence of neutrophils in partial thickness wounds. As in Fig 3B) the differences in MPO positive areas between the treatment groups at the different time points.

In the partial thickness wounds a statistically significant reduction of MPO expression was detected after PBD 8 in all groups (Fig 4A). Although the number of neutrophils seems de be reduced in the treatment groups in comparison to the control at PBD 14 and 22, no statistically significant differences were detected between the treatments except for the Combination therapy at PBD 22 (Fig 5).

### Graft take and epithelialization

**Fig 6:**
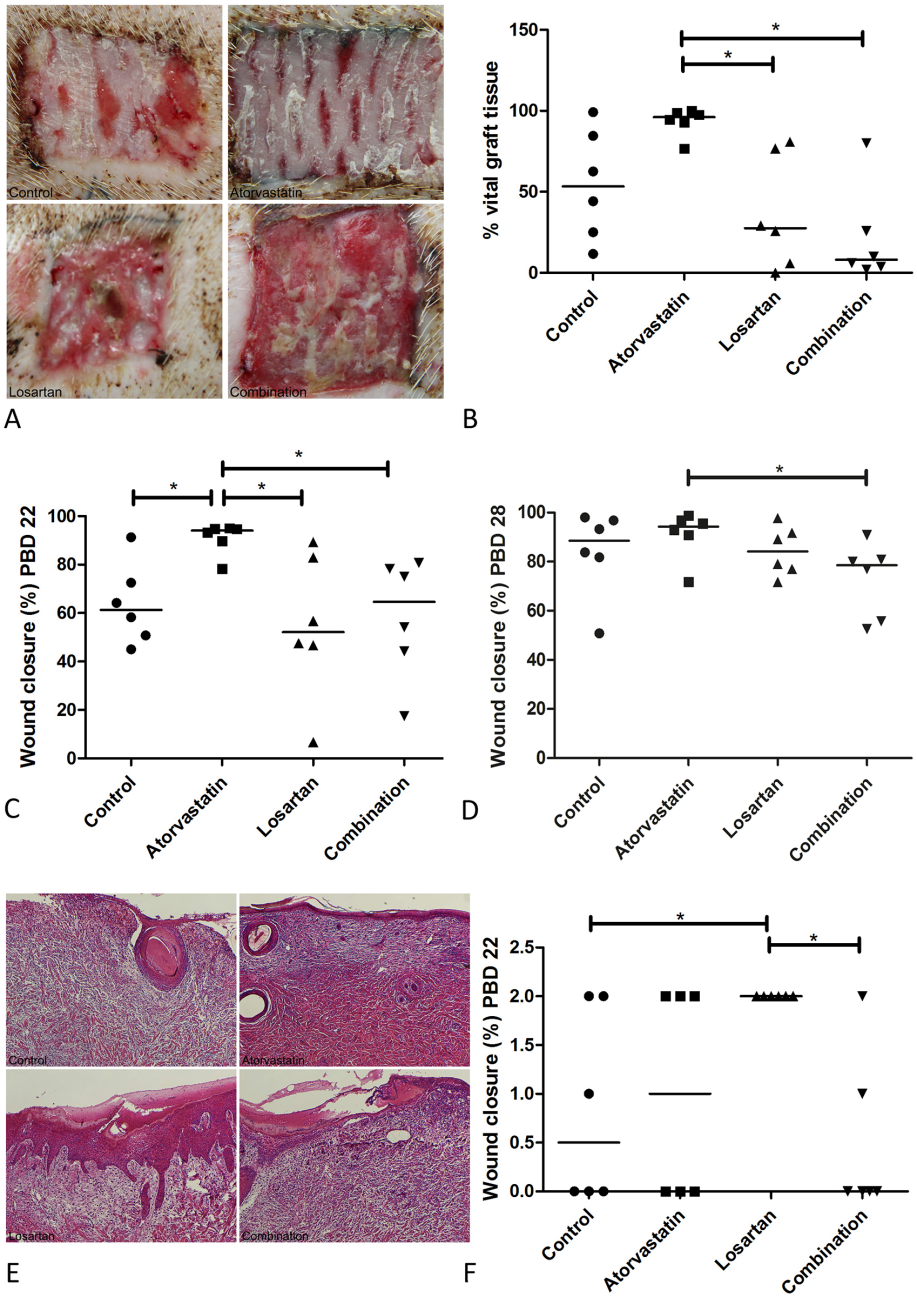
Graft take and re-epithelialization. Fourteen days after infliction of full thickness wounds, the wounds were excised and transplanted with an autologous STSG. The adherence (take) of the graft was macroscopically evaluated at PBD 22. Fig 6A Shows representative picture of the wounds of all treatment groups. In Fig 6B the take of the graft is expressed as percentage of the total graft that adhered to the wound bed and had a pink (viable) appearance. Epithelialization of full thickness wounds was macroscopically evaluated and expressed as percentage of the wound area that was closed at PBD 22 (Fig 6C) and PBD 28 (Fig 6D). Wound closure in partial thickness wounds was assessed histologically on H&E stained slides because the presence of a scab prevented macroscopic analysis. 6E shows representative pictures of the H&E stainings of the different treatments. Fig 6F shows the re-epithelialization scores at PBD 14.

In accordance with clinical practice the necrotic tissue of the full thickness wounds was excised and the wounds were transplanted with autologous STSG, fourteen days after infliction of full thickness wounds (PBD 14). Take of the graft was macroscopically evaluated at PBD 22; Fig 6A shows representative pictures of wounds of the different treatment groups. The percentage of the vital graft present in the wounds is shown in Fig 6B. A better take of the STSG was observed for Atorvastatin treated wounds compared to control, although differences were not statistically significant different due to a high variation in graft take of the control wounds (*p* = 0.055).

In contrast, treatment of full thickness wounds with Losartan or combination therapy caused a significant reduction of the graft take compared to Atorvastatin treatment. Treatments with Losartan alone or in combination with Atorvastatin reduced the take of the STSG compared to the control treatment at PBD 22, but again this difference was not statistically significant (*p* = 0.34 and *p* = 0.055, respectively).

Re-epithelialization of burn wounds within 21 days is known to be associated with better scar outcome, therefore re-epithelialization was evaluated. Full thickness wounds treated with Atorvastatin showed statistically significantly enhanced epithelialization in comparison to all other treatments at PBD 22, 8 days after escharotomy and transplantation with the STSG (Fig 6C). Despite the massive graft loss in the Losartan and combination therapy treated wounds wound closure did not seem affected. No differences in wound closure were observed between the treatments at PBD 28 (Fig 6D).

Since the partial thickness wounds were not excised, it was difficult to see which wounds or parts of the wounds were re-epithelialized macroscopically. Therefore, we scored the H&E sections of the wounds at PBD 14 for the presence of an epidermis. Fig 6E shows representative pictures for the different treatment groups. The partial thickness wounds in the Losartan treated animal showed statistically significantly faster re-epithelialization compared to the control group and combination therapy (Fig 6F), whereas no differences were observed between the other treatment groups.

### Clinical outcome and wound contraction

**Fig 7:**
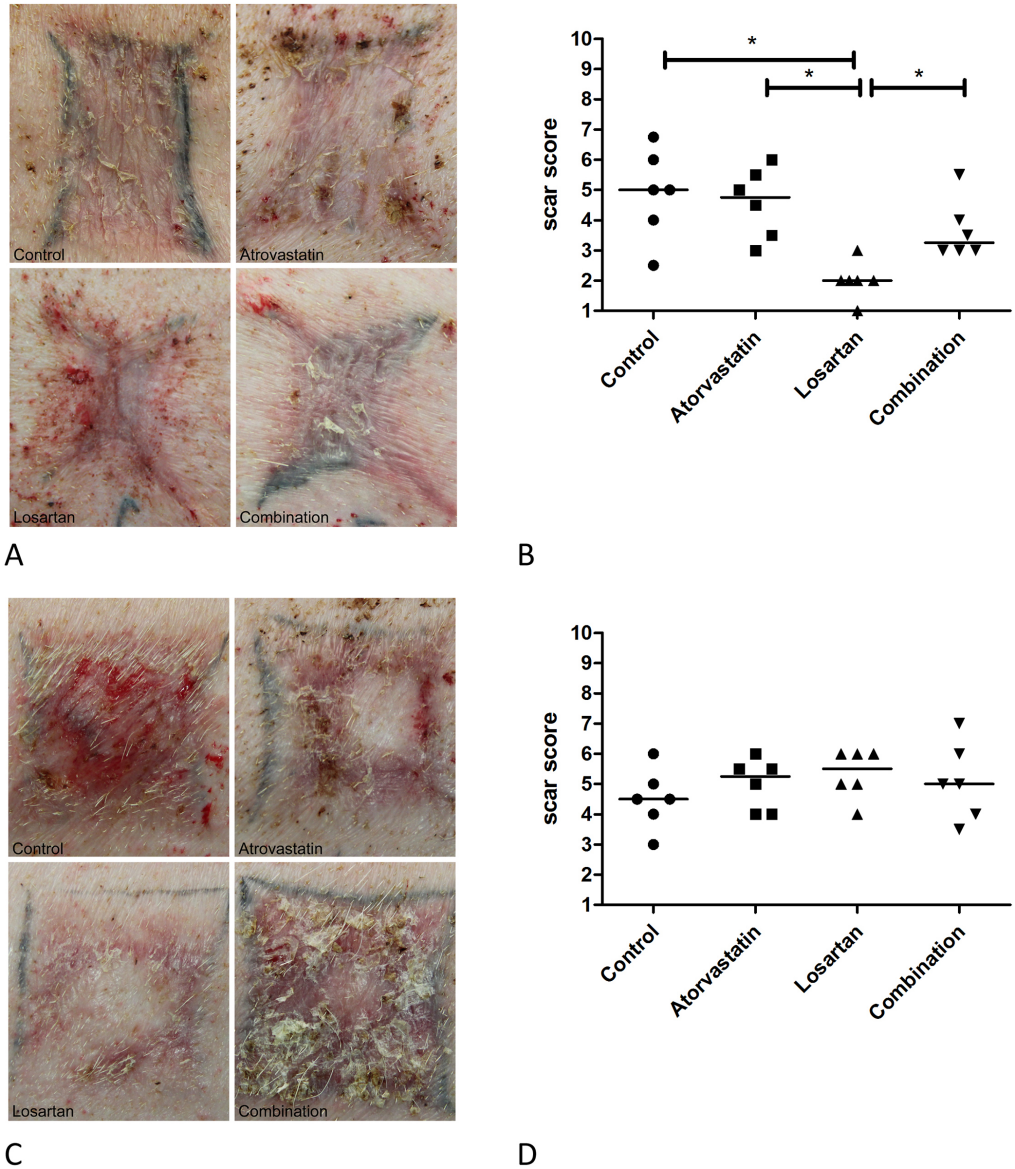
Scar quality. Macroscopic evaluation of scars was performed at PBD 56. Fig 7A and 7C show representative pictures of the scars of full and partial thickness wounds respectively of the different treatment groups. The results of the observer scores given for each treatment for the full thickness wounds (7B) and partial thickness wounds (Fig 7C) are shown. Normal, uninjured skin is scored 1 and the poorest outcome imaginable receives the grade 10.

Scar quality was assessed macroscopically at PBD 56. Scar quality was quantified by means of an adapted observer part of the POSAS scale. Fig 7A and 7C shows representative pictures of the scars from the full and partial thickness wounds respectively from all treatment groups. Fig 7B shows the scar scores for the full thickness wounds; a lower score means worse scar outcome. In contrast to the similar scar outcomes of control and Atorvastatin, scars from Losartan-treated wounds had a statistically significant worse observer score. In addition, the scars of the full thickness wounds treated with the combination therapy showed an intermediate scar quality. In the partial thickness wounds all treatments resulted in similar scar scores (Fig 7D).

**Fig 8:**
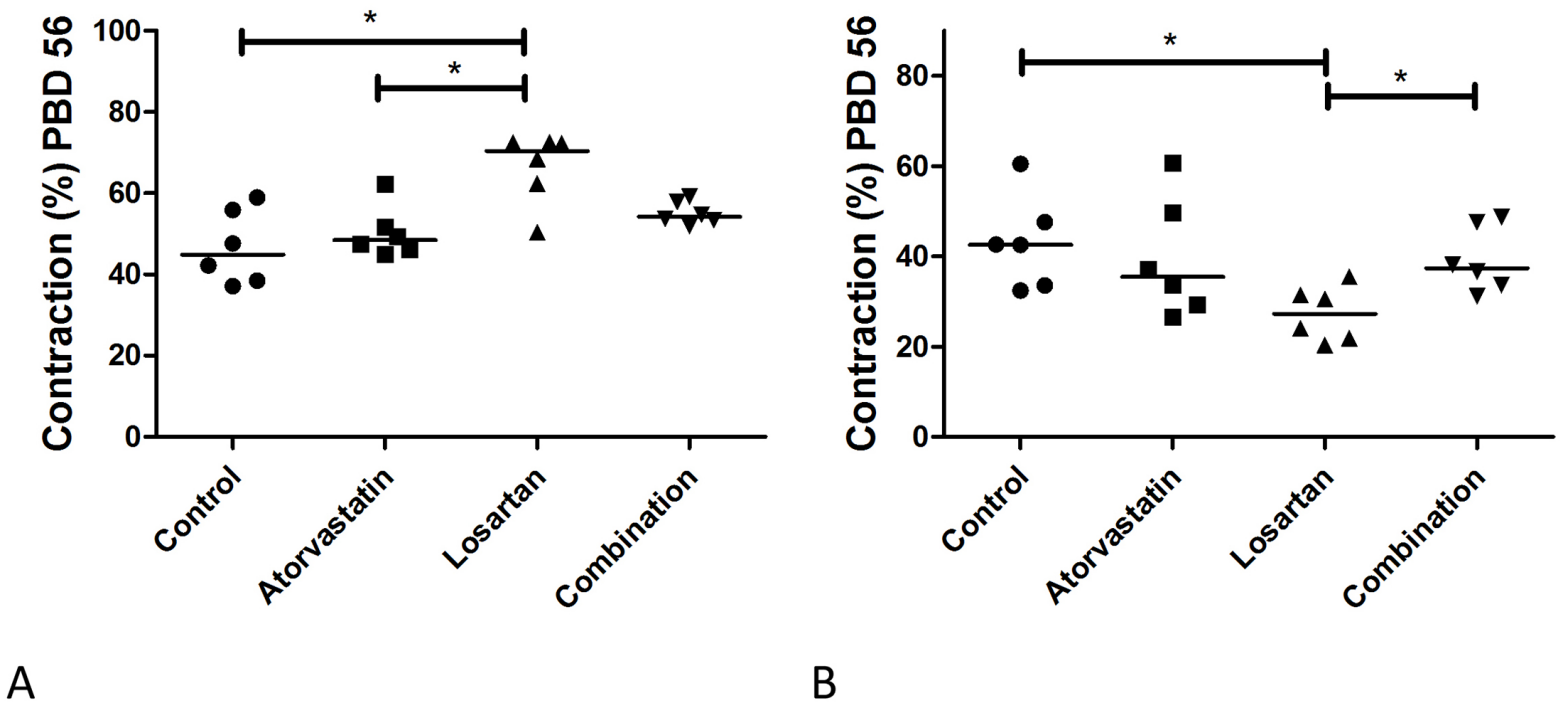
Contraction. Fig 8 Contraction of the full (A) and partial (B) thickness wounds was assessed by planimetry at PBD 56.

Contraction is a major contributor to scar quality and clinical outcome. In this study, control and Atorvastatin treated full thickness wounds showed similar results regarding wound contraction at PBD 56. The Losartan treated full thickness wounds showed the highest contraction compared to control and Atorvastatin treatment (Fig 8A). This was also reflected by a more star-shaped scar (Fig 7A).

Atorvastatin in the combination therapy seemed to counteract the effect of Losartan on contraction of the scars despite the very low graft take in these wounds. However the difference between the combination therapy and losartan treat group did not reach statistical significance (p = 0.055). In partial thickness wounds the Losartan treatment showed a statistically significant reduced contraction in comparison to control and combination therapy (Fig 8B), which might be related to the high re-epithelialization rate (Fig 6D).

**Fig 9:**
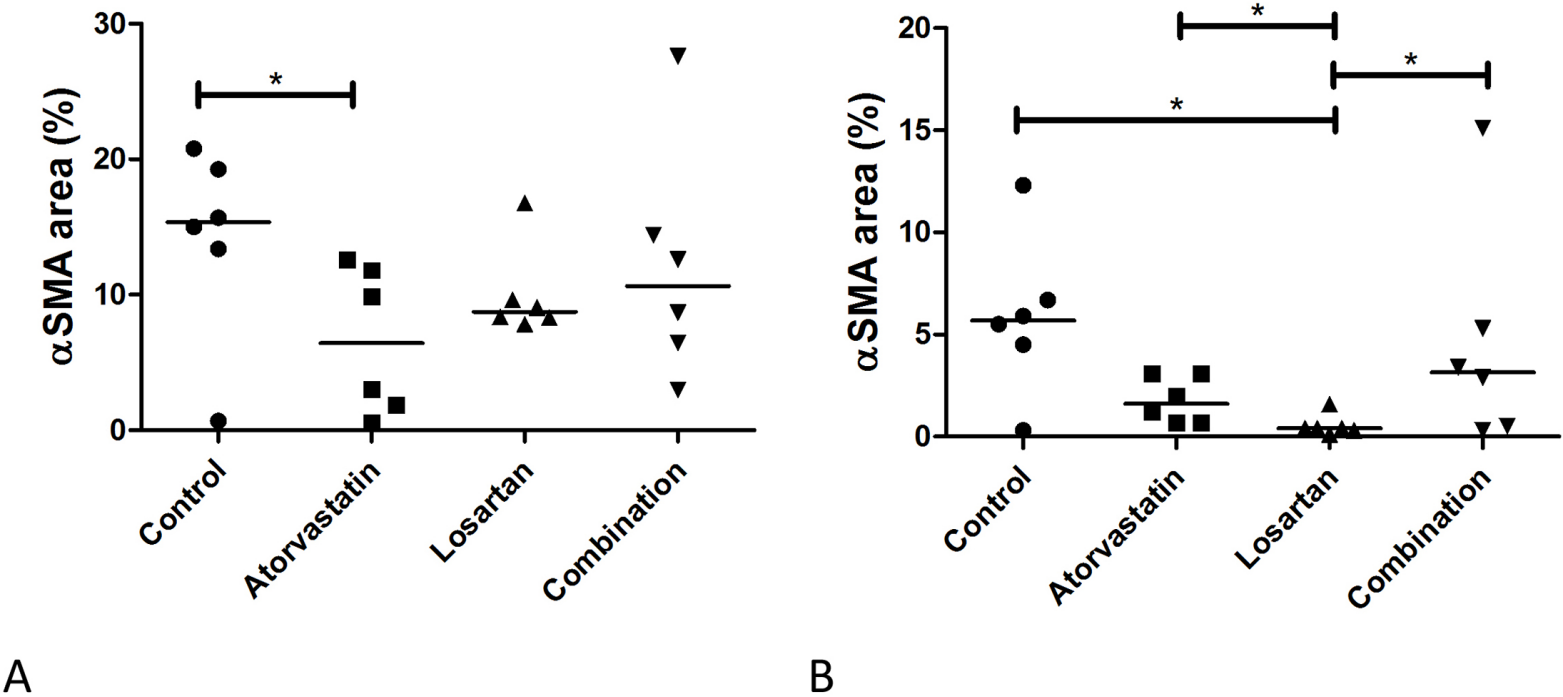
Presence of myofibroblasts in the wound area. Presence of myofibroblasts in wound areas of PBD 28, 35 and 56 was investigated by immunohistochemical staining of αSMA (A). Fig 9B (full thickness wounds) and 9C (partial thickness wounds) show the comparison of αSMA at PBD 56 between the different treatment groups.

Myofibroblasts, characterized by αSMA expression, are crucial for tissue regeneration but are also responsible for overproduction of ECM proteins during fibrosis and contraction. In the full thickness wounds the Atorvastatin treatment showed statistically significant decreased αSMA-positive expression wounds compared to the control treatment at PBD 56 (Fig 9A).

In the partial thickness wounds the Losartan treatment resulted in reduced myofibroblasts in comparison to all other groups at PBD 56 (Fig 9B).

## Discussion

Promising therapeutic effects of AT1 antagonists and statins have been reported on the reduction of fibrosis in different organs. However, the effects of these drugs on healing of partial and full thickness burn wounds have not been described yet. We studied the effects of Losartan, Atorvastatin and a combination of both drugs on the healing of full and partial thickness burn wounds in a porcine model.

The inflammatory reaction is the first phase in the wound healing cascade and necessary to clear the wound from invading micro-organisms and damaged tissue. In addition, the inflammatory reaction recruits different cell types necessary to restore the integrity of the skin.

Neutrophils are the first inflammatory cells to enter the wound environment. Besides their role in preventing infection these cells are also responsible for the attraction of other immune cells [32]. Resolution of the neutrophil influx is crucial for the transition to the next phase of the wound healing process: the proliferative phase. Excessive and prolonged influx of neutrophils can lead to extra tissue damage, enhanced inflammation and delayed wound healing [33]. High numbers and prolonged presence of neutrophils have been observed in human burn wounds. Depletion of neutrophils enhances re-epithelialization and is thought to decrease scar formation [33, 34]. In the literature several studies reported a reduction of the inflammation reaction by statin treatment [35, 36]. Recently it was shown that statins can skew macrophage differentiation towards the anti-inflammatory M2 macrophage. M2 macrophages are thought to play a role in the resolution of the inflammatory reaction [37, 38]. In this study we saw a faster resolution of neutrophils with Atorvastatin treatment in full thickness burn wounds. The number of neutrophils was significantly lower in the Atorvastatin group in comparison with the other treatments at PBD 14, the day of eschar excision and split skin transplantation. Whether this was mediated by M2 macrophages could not be determined in this study since there are no specific antibodies to discriminate between different macrophages subtypes in the pig.

We did not find an effect of either treatment on neutrophils in partial thickness wounds, but in these wounds the number of neutrophils was much lower than in the full thickness wounds, indicating a milder inflammatory reaction.

We further demonstrated that Atorvastatin treatment enhanced graft take and re-epithelialization, which may be related to the reduced inflammation and improved vascularization of the wound bed. Wang et al. showed that Atorvastatin enhanced angiogenesis and vascular maturation [39].

Atorvastatin treatment also resulted in faster resolution of myofibroblast presence in the wound, however, this did not result in a better scar score or significant reduction in contraction. In contrast to the Atorvastatin treatment, Losartan treatment reduced the graft take resulting in a diminished scar outcome and more contraction compared to the control group. The reduced graft take did not disturb wound closure 8 days after transplantation (PBD 22), indicating that migration and proliferation of keratinocytes were not affected but that the poor outcome is likely related to the loss of the dermal part of the graft. The epidermal stem cells residing in the hair follicles are known to play an important role in re-epithelialization and it was shown that the absence of hair follicle delays re-epithelialization [40]. Since in the full thickness wounds the hair follicles are completely destroyed, the epidermal cells covering the wounds migrate into the wound area from the wound edges and from the split skin autograft. In the Losartan treated wounds the migration of epidermal cells from the graft and the wound edges probably was not disturbed despite the reduced graft take.

In partial thickness wounds part of the hair follicle is still present and viable. Losartan seemed to increase re-epithelialization of the partial thickness wounds probably by stimulating proliferation and migration of the hair follicle stem cells. These results are in agreement with Kamber et al who also showed enhanced re-epithelialization upon Losartan treatment in a diabetic wound model in mice [41]. In contrast, Takeda et al showed that Candesartan (another AT1 antagonist) treatment reduced re-epithelialization in a rat full thickness wound model [42]. The discrepancy between our study and the latter study may be the dose of the AT1 antagonist: in our study we used the recommended human dose for both drugs, whereas Tadeka et al used doses 10 and 100 times higher than the recommended dose.

Beside re-epithelialization rebuilding the lost dermis is important in restoring the integrity of the skin. Myofibroblasts, characterized by the presence of αSMA containing stress fibers, play an important role in this part of the wound healing process via the synthesis of extracellular matrix components. Fibrosis, as seen in hypertrophic scars, is associated with the prolonged activation and excessive presence of myofibroblasts. Atorvastatin treatment of full thickness wounds showed at first an increase in αSMA positive wound area at PBD 28, which was significantly higher than the αSMA positive area of the control group. However, the αSMA-positive area reduced gradually over time in the Atorvastatin group, whereas the αSMA-positive area fractions of the wounds of control, Losartan and combination therapy remained at similar high levels during the entire study period.

In the partial thickness wounds Losartan treatment resulted in a significant decrease of myofibroblasts, which is in agreement with the αSMA lowering effects of AT1 antagonists observed in other fibrotic pathologies [43–46]. To our knowledge the effects of Losartan on the fibrotic processes in burn wound healing has not been studied earlier. However, in other models of tissue injury administration of Losartan exerted anti-fibrotic actions when started at later time points after induction of injury compared to administration directly after the defect was created [43, 47]. Data of these studies indicate that timing of Losartan administration seems to be important for the therapeutic effects of Losartan. In our study, the proposed anti-fibrotic functions of AT1 antagonists in full thickness wounds might have been more effective if the therapy had started at a later time point; e.g. after the graft survival. The results of our study suggest that downstream actions of the AT1 are important for a successful graft take on full thickness wounds. Therefore, additional research is needed to determine the optimal timing of administration Losartan.

In summary, we showed that the combination of AT1 inhibition with statin treatment does not improve wound healing, but that the used drugs exert different functions in partial and full thickness burn wounds. Characteristics of the (remaining) wound tissue and extent of the pro-inflammatory phase in full and partial thickness burn wounds might be involved in the dualistic response to Losartan and Atorvastatin.

Atorvastatin possibly speeds up the wound healing process in full thickness burn wounds by improving graft take, promoting an earlier transition from the inflammatory to the proliferative phase and subsequently by stimulating a faster resolution of myofibroblasts. On the other hand, Losartan seems to improve keratinocyte migration and proliferation thereby speeding up wound closure.

The findings in this study are promising but further research is needed. For instance the timing of Losartan treatment might be crucial; administration of the drug after graft survival might improve the healing process of full thickness burn wounds as well.

## Supporting information

S1 Table All data full thickness wounds. (PDF)

S2 Table All data partial thickness wounds. (PDF)

## Abbreviations

αSMA: alpha-smooth muscle actin
ACE: angiotensin converting enzyme
AngII: angiotensin II
AT1: angiotensin receptor 1
AT2: angiotensin receptor 2
DAB: 3,3'-Diaminobenzidine
ECM: extracellular matrix proteins
IL-6: interleukin-6
MPO: myeloperoxidase
PBD: post-burn day
PBS: phosphate buffered saline
POSAS: patient and observer scar assessment scale
RAS: renin angiotensin system
ROI: region of interest
ROS: reactive oxygen species
STSG: split-thickness skin graft
TGF-ß1: transforming growth factor beta-1
TNF-α: tumor necrosis factor-alpha
tRAS: tissue renin angiotensin system
