# Supplementary material for: Differential effects of Losartan and Atorvastatin in partial and full thickness burn wounds
Source: PLoS One. 2017 Jun 14;12(6):e0179350. doi: 10.1371/journal.pone.0179350 (PMC5470692; doi:10.1371/journal.pone.0179350)
Supplement: S1 Table — (PDF) [file pone.0179350.s001.pdf]

treatment	aSMA	MPOd8	MPOd14	MPOd22	MPOd28	Wound_closure
Atorvastatin	1,90	3,07	0,57	0,10	0,11	94,83
Atorvastatin	11,80	2,33	0,77	0,18	0,01	89,83
Atorvastatin	12,60	7,60	0,17	0,14	0,00	78,33
Atorvastatin	3,00	2,53	0,44	0,11	0,01	95,00
Atorvastatin	0,55	2,40	0,42	0,04	0,03	93,33
Atorvastatin	9,85	3,13	0,19	0,07	0,01	94,67
Losartan	9,65	3,63	1,36	0,02	0,03	46,67
Losartan	7,85	4,50	0,86	2,04	0,08	89,33
Losartan	8,40	3,83	0,53	1,54	0,05	82,92
Losartan	16,80	0,75	1,20	0,14	0,08	56,67
Losartan	9,05	0,17	1,08	1,37	0,12	47,50
Losartan	8,30	3,17	1,69	2,31	1,44	6,67
combination therapy	2,95	2,84	1,01	1,07	0,07	54,17
combination therapy	14,40	2,20	0,86	0,05	0,09	78,33
combination therapy	6,45	0,34	0,66	0,17	0,02	80,83
combination therapy	8,70	1,13	0,98	0,14	0,77	17,50
combination therapy	27,60	1,10	2,47	1,04	0,50	44,17
combination therapy	12,60	1,27	3,44	0,07	0,01	75,00
control	0,70	3,33	1,76	0,04	0,15	72,50
control	20,80	2,83	1,48	0,07	0,02	58,33
control	15,00	0,20	4,35	0,57	0,10	50,83
control	19,25	0,43	0,52	0,10	0,01	91,33
control	13,40	0,05	0,67	2,00	0,01	45,00
control	15,70	2,04	1,77	0,07	0,02	64,17

treatment	scar_score	graft_take	contraction	MPOatorvastatin	MPOLosartan
Atorvastatin	5,00	100,00	47,50	3,07	3,63
Atorvastatin	4,50	92,92	51,68	2,33	4,50
Atorvastatin	3,00	76,67	62,30	7,60	3,83
Atorvastatin	6,00	97,50	46,20	2,53	0,75
Atorvastatin	5,50	94,58	45,07	2,40	0,17
Atorvastatin	3,50	98,83	49,36	3,13	3,17
Losartan	2,00	29,17	72,54	0,57	1,36
Losartan	2,00	80,83	68,45	0,77	0,86
Losartan	2,00	76,67	72,25	0,17	0,53
Losartan	2,00	25,83	62,38	0,44	1,20
Losartan	3,00	5,83	50,40	0,42	1,08
Losartan	1,00	0,00	72,45	0,19	1,69
combination therapy	5,50	3,83	54,70	0,10	0,02
combination therapy	3,00	25,83	53,73	0,18	2,04
combination therapy	3,50	10,00	53,39	0,14	1,54
combination therapy	3,00	2,00	59,16	0,11	0,14
combination therapy	3,00	6,00	51,99	0,04	1,37
combination therapy	4,00	80,00	57,86	0,07	2,31
control	6,75	84,50	38,46	0,11	0,03
control	5,00	62,50	37,12	0,01	0,08
control	5,00	44,17	58,92	0,00	0,05
control	6,00	99,17	42,19	0,01	0,08
control	2,50	11,67	47,61	0,03	0,12
control	4,00	25,00	55,85	0,01	1,44

treatment	MPOcombination	MPOcontrol	timepoints
Atorvastatin	2,84	3,33	8,00
Atorvastatin	2,20	2,83	8,00
Atorvastatin	0,34	0,20	8,00
Atorvastatin	1,13	0,43	8,00
Atorvastatin	1,10	0,05	8,00
Atorvastatin	1,27	2,04	8,00
Losartan	1,01	1,76	14,00
Losartan	0,86	1,48	14,00
Losartan	0,66	4,35	14,00
Losartan	0,98	0,52	14,00
Losartan	2,47	0,67	14,00
Losartan	3,44	1,77	14,00
combination therapy	1,07	0,04	22,00
combination therapy	0,05	0,07	22,00
combination therapy	0,17	0,57	22,00
combination therapy	0,14	0,10	22,00
combination therapy	1,04	2,00	22,00
combination therapy	0,07	0,07	22,00
control	0,07	0,15	28,00
control	0,09	0,02	28,00
control	0,02	0,10	28,00
control	0,77	0,01	28,00
control	0,50	0,01	28,00
control	0,01	0,02	28,00
