# Supplementary material for: Differential effects of Losartan and Atorvastatin in partial and full thickness burn wounds
Source: PLoS One. 2017 Jun 14;12(6):e0179350. doi: 10.1371/journal.pone.0179350 (PMC5470692; doi:10.1371/journal.pone.0179350)
Supplement: S2 Table — (PDF) [file pone.0179350.s002.pdf]

treatment	aSMAd56	MPOd8	MPOd14	MPOd22	epidermis	scar_score
Atorvastatin	0,70	6,62	0,04	0,01	2,00	5,50
Atorvastatin	0,70	0,90	0,01	0,01	0,00	5,50
Atorvastatin	3,10	0,06	0,07	0,01	0,00	6,00
Atorvastatin	2,00	0,75	0,02	0,07	2,00	5,00
Atorvastatin	1,20	5,33	0,12	0,03	0,00	4,00
Atorvastatin	3,10	7,89	0,05	0,02	2,00	4,00
Losartan	0,30	0,40	0,02	0,03	2,00	6,00
Losartan	0,10	2,33	0,11	0,01	2,00	5,00
Losartan	0,40	0,02	0,07	0,02	2,00	4,00
Losartan	0,40	2,46	0,04	0,01	2,00	6,00
Losartan	0,40	1,90	0,04	0,01	2,00	6,00
Losartan	1,60	0,28	0,01	0,01	2,00	5,00
combination therapy	0,50	4,38	0,13	0,04	1,00	5,00
combination therapy	5,30	0,31	0,08	0,00	0,00	5,00
combination therapy	0,30	0,20	0,00	0,00	2,00	7,00
combination therapy	2,90	0,05	0,10	0,00	0,00	4,00
combination therapy	3,40	3,15	0,14	0,01	0,00	6,00
combination therapy	15,10	3,71	0,20	0,04	0,00	3,50
control	4,50	4,88	0,02	0,01	2,00	4,50
control	5,50	2,17	0,28	0,01	0,00	4,50
control	0,30	0,30	0,07	0,07	2,00	5,00
control	5,90	0,02	0,18	0,08	0,00	4,00
control	6,70	6,46	0,24	0,07	1,00	6,00
control	12,30	0,26	0,26	0,10	0,00	3,00

treatment	contraction	MPOatorvastatin	MPOLosart	MPOcombination	MPOcontrol
Atorvastatin	33,67	6,62	0,40	4,38	4,88
Atorvastatin	49,78	0,90	2,33	0,31	2,17
Atorvastatin	29,31	0,06	0,02	0,20	0,30
Atorvastatin	37,13	0,75	2,46	0,05	0,02
Atorvastatin	60,70	5,33	1,90	3,15	6,46
Atorvastatin	26,66	7,89	0,28	3,71	0,26
Losartan	30,56	0,04	0,02	0,13	0,02
Losartan	31,46	0,01	0,11	0,08	0,28
Losartan	21,84	0,07	0,07	0,00	0,07
Losartan	20,33	0,02	0,04	0,10	0,18
Losartan	35,48	0,12	0,04	0,14	0,24
Losartan	24,01	0,05	0,01	0,20	0,26
combination therapy	48,74	0,01	0,03	0,04	0,01
combination therapy	47,56	0,01	0,01	0,00	0,01
combination therapy	31,13	0,01	0,02	0,00	0,07
combination therapy	38,11	0,07	0,01	0,00	0,08
combination therapy	33,63	0,03	0,01	0,01	0,07
combination therapy	36,63	0,02	0,01	0,04	0,10
control	42,53				
control	47,60				
control	33,54				
control	42,64				
control	32,41				
control	60,52				

treatment	timepoints
Atorvastatin	8,00
Atorvastatin	8,00
Atorvastatin	8,00
Atorvastatin	8,00
Atorvastatin	8,00
Atorvastatin	8,00
Losartan	14,00
Losartan	14,00
Losartan	14,00
Losartan	14,00
Losartan	14,00
Losartan	14,00
combination therapy	22,00
combination therapy	22,00
combination therapy	22,00
combination therapy	22,00
combination therapy	22,00
combination therapy	22,00
control	
control	
control	
control	
control	
control	
